# Supplementary material for: CircCamsap1 is dispensable for male fertility in mice
Source: PeerJ. 2024 May 21;12:e17399. doi: 10.7717/peerj.17399 (PMC11122046; doi:10.7717/peerj.17399)
Supplement: Supplemental Information 1 [file peerj-12-17399-s001.pdf]

Table S1. Primers used in PCR,RT-PCR and RT-qPCR

Primer name	Primer sequence(5'-3')	Expected product size (bp)
Convergent-F	CTGACCTCAGCCAGGCACCTATT	291
Convergent-R	GCTCTCGCCGATAACGGACCTT	
Divergent-F	TCTGGATCAATAAGACAACATCCCTG	124
Divergent-R	ACGGCAATACAGCTCACTGGACAG	
circCamsap1-/--5+3 arm-F	GGTTGCTATGTTCTCAGAGGCTAT	1208
circCamsap1-/--5+3 arm-R	CTCAGGCTACATTGCTTCCTTACT	
CacircCamsap1-/--5 arm-F	GCACTCAGCCAGAATCGGTTG	1061
circCamsap1-/--5 arm-R	CCAGCACAGCAGCAATCTCAG	
circCamsap1-/--3 arm-F	ATGGTGGTGAAGTGGAGATATAGG	1303
circCamsap1-/--3 arm-R	GAAGCAGGAAGGAAGGTGGAA	
circCamsap1-RT-(Q)pcr-F	ATGGTGGATGCCCTGATGATGG	214
circCamsap1-RT-(Q)pcr-R	GGCTTAATGTGCTCCTGCTCATAC	
Camsap1-RT-qpcr-F	CTGACCTCAGCCAGGCACCTATT	291
Camsap1-RT-qpcr-R	GCTCTCGCCGATAACGGACCTT	
18S-F	TAACGAACGAGACTCTGGCAT	138
18S-R	CGGACATCTAAGGGCATCACAG	
