# Supplementary figures and images for: CircCamsap1 is dispensable for male fertility in mice
Source: PeerJ. 2024 May 21;12:e17399. doi: 10.7717/peerj.17399 (PMC11122046; doi:10.7717/peerj.17399)

Figure 1b

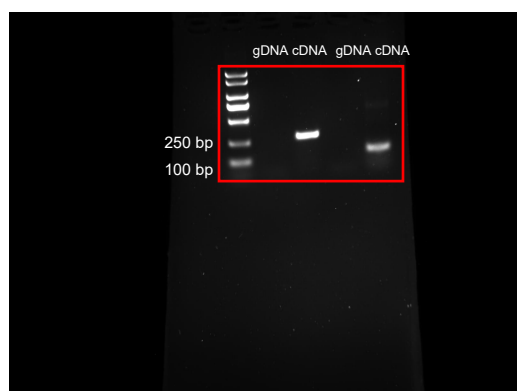

Figure 2b

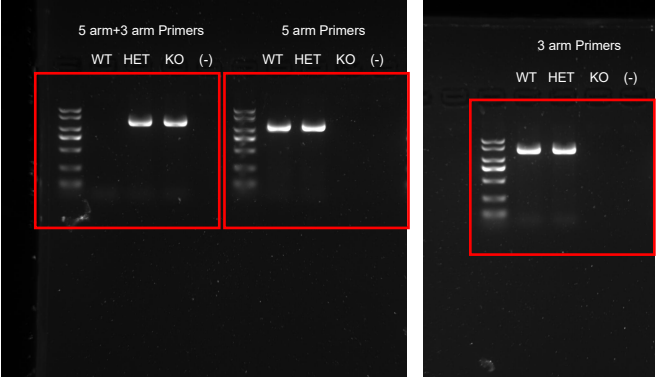

Figure 2c

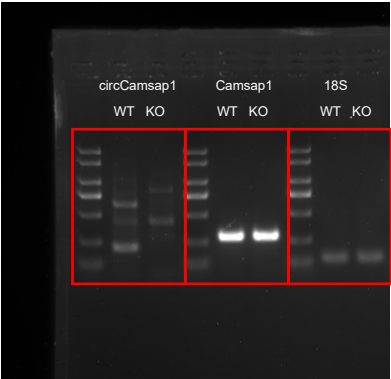

Supplement: Supplemental Information 3 [file peerj-12-17399-s003.pdf]
